# Assessment of peripheral biomarkers potentially involved in episodic and chronic migraine: a case-control study with a focus on NGF, BDNF, VEGF, and PGE2

**DOI:** 10.1186/s10194-021-01377-6

**Published:** 2022-01-06

**Authors:** Mohammad Mozafarihashjin, Mansoureh Togha, Zeinab Ghorbani, Abolfazl Farbod, Pegah Rafiee, Fahimeh Martami

**Affiliations:** 1 Headache Department, Iranian Center of Neurological Research, Neuroscience Institute, Tehran University of Medical Sciences, Tehran, Iran; 2 Lunenfeld-Tanenbaum Research Institute, Sinai Health System, Toronto, Ontario Canada; 3 Headache Department, Neurology Ward, School of Medicine, Sina University Hospital, Tehran University of Medical Sciences, Tehran, Iran; 4 Cardiovascular Diseases Research Center, Department of Cardiology, School of Medicine, Heshmat Hospital, Guilan University of Medical Sciences, Rasht, Iran; 5 Department of Clinical Nutrition, School of Medicine, Guilan University of Medical Sciences, Rasht, Iran; 6 Department of Clinical Nutrition and Dietetics, Faculty of Nutrition Sciences and Food Technology, National Nutrition and Food Technology Research Institute, Shahid Beheshti University of Medical Sciences, Tehran, Iran; 7 School of nutritional Sciences and Dietetics, Tehran University of Medical Sciences, Tehran, Iran

**Keywords:** Headache, Migraine, Biomarker, Nerve growth factor, Brain-derived neurotrophic factor, Vascular endothelial growth factor, Prostaglandin E2

## Abstract

**Background:**

Several inflammatory and vascular molecules, and neurotrophins have been suggested to have a possible role in the development of migraine. However, pathophysiological events leading to migraine onset and transformation of episodic migraine (EM) to chronic migraine (CM) are not fully understood. Thus, we aimed to assess peripheral levels of nerve growth factor (NGF), brain-derived neurotrophic factor (BDNF), vascular endothelial growth factor (VEGF), and prostaglandin E2 (PGE2) in EM and CM patients, and controls.

**Methods:**

From September 2017 to June 2020, 89 subjects were enrolled in a case-control study; 23 and 36 EM and CM patients, respectively, and 30 age and sex-matched controls. Demographic data and medical history were obtained from all patients. Headache characteristics were recorded at baseline visit and ensuing 30 days for persons with migraine disease. Serum levels of NGF, BDNF, VEGF, and PGE2 were measured once for controls and EM and CM patients, and adjusted for age, sex, and body mass index.

**Results:**

Serum levels of NGF were significantly lower in EM patients compared to controls and CM patients (*P-*value=0.003 and 0.042, respectively). Serum levels of BDNF were significantly lower in EM and CM patients as opposed to controls (*P-*value<0.001), but comparable between EM and CM patients (*P*-value=0.715). Peripheral blood levels of VEGF were significantly higher in EM and CM patients as opposed to controls (*P-*value<0.001), but not different between EM and CM patients (*P*-value=0.859). Serum levels of PGE2 were significantly lower in EM patients compared to controls (*P*-value=0.011), however similar between EM and CM patients (*P-*value=0.086). In migraine patients, serum levels of NGF and PGE2 positively correlated with headache frequency (NGF: ρ = 0.476 and *P-*value<0.001; PGE2: ρ = 0.286 and *P-*value=0.028), while corresponding levels of BDNF and VEGF did not correlate with headache frequency (BDNF: ρ = 0.037 and *P-*value=0.778; VEGF: ρ= -0.025 and *P-*value=0.850).

**Conclusions:**

Our findings suggest that NGF, BDNF, PGE2, and VEGF may play a significant role in migraine pathogenesis and/or chronification, and therefore might bear potential value for novel targeted abortive and prophylactic migraine therapy. Further prospective cohort studies with larger sample sizes can more robustly evaluate the implications of these findings.

**Supplementary Information:**

The online version contains supplementary material available at 10.1186/s10194-021-01377-6.

## Background

Migraine headache is a disabling neurological disorder that carries with it a significant burden which crosses over multiple domains on both personal and societal levels, including the negative impact on work productivity, family life, mental health, leisure activities, and the economy [1–3]. The yearly prevalence of migraine is close to 15% in the general public. A systematic analysis of the 2016 global burden disease (GBD) study estimated that just above one billion people in the world had migraine in 2016 [4]. Based on the latest GBD study in 2019, migraine headache is the second most frequent cause of global disability in all age groups combined (regardless of sex), and first most frequent cause of global disability in 15-49 year-old women, specifically [5]. Persons with migraine disease suffer from either episodic migraine (EM), defined as recurrent headache disorder manifesting in at least five attacks lasting 4–72 h with typical characteristics, or chronic migraine (CM), diagnosed when the individual has headache occurring on 15 or more days/ month for more than three months, which, on at least eight days/month, has the features of migraine headache [6–8].

Both environmental and genetic factors have been implicated in the development of migraine [6, 9]. The pathophysiology of migraine is not fully understood, and multiple theories have been propounded to explain this pathogenesis including trigeminovascular pathway activation, vascular dysfunction, cortical spreading depression (CSD), and neuroinflammation [6, 10, 11]. Migraine attacks have been suggested to have either a peripheral origin, in which first order trigeminovascular neurons are activated, or a central origin, where CSD and hypothalamic/brainstem activation play a major role [9]. Pre-clinical data have suggested that during a migraine attack, neuropeptides such as calcitonin gene-related peptide (CGRP) and pituitary adenylate cyclase-activating peptide (PACAP) are increasingly released from afferent nociceptive fibers and act on vascular smooth muscle cells of meningeal arteries to trigger a cascade of intracellular signaling events mediated by cyclic adenosine monophosphate and cyclic guanosine monophosphate that ultimately lead to opening of potassium channels and vasodilation [7, 9]. In addition, the release of CGRP and PACAP can lead to degranulation of meningeal mast cells, which in turn can increase levels of proinflammatory mediators such as prostaglandin E2 (PGE2) [12, 13]. It has also been proposed that CSD can trigger mast cells to excrete such proinflammatory molecules [14]. PGE2 enhances inflammatory pain and possibly nociceptor sensitization, and can itself increase the release of CGRP in preclinical models [13, 15]. Moreover, the endothelial cells of the meningeal arteries can also release vascular endothelial growth factor (VEGF) that increases vascular leakage, nitric oxide (NO) synthesis, and mobilizes more macrophages and neutrophils to adjacent tissues [16, 17]. These immune cells excrete cytokines that promote neuronal sensitization [16]. In clinical models of migraine, infusion of CGRP, PACAP, PGE2, or glyceryl trinitrate appeared to provoke headache attacks [7, 9, 13]. Also, studies have indicated that neurotrophins such as nerve growth factor (NGF) and brain-derived neurotrophic factor (BDNF) may have a role in modulating nociceptive pathways [18]. To be specific, inflammation could give rise to increased production of NGF which can in turn increase the expression of pain receptors such as transient receptor potential vanilloid receptor 1 (TRPV1) on peripheral nociceptive fibers and the excretion of CGRP and BDNF [18]. Other mechanisms that have been implicated in migraine are increased sensitivity to NO, serotonin secretion from platelets, increased levels of homocysteine, decreased vitamin D levels, and oxidative stress [6, 11, 19–23]. Although the effectiveness of various prescribed medications in dampening migraine symptoms is generally acceptable, adverse effects such as gaining or losing weight, drop in blood pressure, decreased awareness, and sleepiness/lethargy restrict the use of such treatments. Moreover, suboptimal treatment of EM in persons with migraine disease can facilitate the transformation of EM to CM, which is a more burdensome state [6]. Therefore, identifying more tolerable and efficacious options is indispensable [24–26]. On the other hand, probing for biomarkers such as neurotransmitters, receptors, and inflammatory factors can potentially reveal mechanisms involved in development of migraine, provoking the attacks, and evolution of CM, and promote the development of novel and more effective anti-migraine medications. Taking this into account, we aimed to investigate potential biomarkers associated with migraine in EM and CM patients with a special focus on PGE2, VEGF, NGF and BDNF.

## Methods

### Study population

From September 2017 to June 2020, we recruited study participants for a case-control study in Tehran, Iran through an advertisement (posters mainly placed in headache clinic and the immediate vicinity but also all over the hospital) to evaluate the role of several biomarkers in migraine. Cases were EM and CM patients who were selected from a tertiary headache clinic at Sina Hospital (affiliated to Tehran University of Medical Sciences), and age and sex-matched healthy controls (i.e., not suffering from headaches in general) were enrolled from the general population (hospital staff and patient companions). All potential participants underwent a medical evaluation by a clinical neurologist sub-specialized in headache disorders to confirm the diagnosis of EM and CM as per diagnostic criteria outlined in the third edition of the international classification of headache disorders [8]. Cases were included if their primary diagnosis of EM/CM was made at least six months prior to study commencement. To be eligible for the study, participants had to have been aged between 18 and 65 years, have a body mass index (BMI) between 18.5 and 35 kg/m^2^, have not been diagnosed simultaneously with medication overuse headache (MOH), and not have a medical history positive for any of the following: infectious, cardiovascular, or endocrinological diseases, allergic, immunological, renal and hepatic disorders, and other chronic neurological diseases such as Parkinson’s disease, multiple sclerosis, epilepsy, or Alzheimer’s disease. Subjects were excluded if they were pregnant or breastfeeding. Following an adequate explanation of study rationale, procedures, and goals, all study participants provided written informed consent before the start of the study. The ethical committee of National Institute for Medical Research Development (NIMAD) approved this study (ID: IR.NIMAD.REC.1396.054).

### Data collection

During the initial study visit, study participants were interviewed to collect relevant demographic data, and capture past medical history and best possible medication history. Weight and height of all study participants were measured via Seca Clara 803 digital scale (accuracy of 0.01 g; Seca GmbH & Co. KG., Hamburg, Germany) and Seca 216 wall-mount stadiometer (accurate to 0.1 cm without shoes; Seca GmbH & Co. KG., Hamburg, Germany), and BMI was calculated as weight (in kg)/(height (in metre))^2^. Peripheral venous blood samples were collected from controls at this timepoint. Participants in the case arm of the study underwent a medical evaluation conducted by the study neurologist/headache sub-specialist (M.T.) to confirm their initial migraine diagnosis. Then, cases were given a daily headache diary (designed by M.T. [27]) and instructed on how to use the diary to record the characteristics of any headaches they experienced in the ensuing 30 days. The study staff also followed up with the participants via telephone on a weekly basis over this month. Data captured through this diary included the intensity, duration (time elapsed from headache onset to cease of headache by itself or through abortive medications, whichever is sooner) and frequency (i.e., number of headache days per month) of headaches, type of abortive/analgesic medications taken, and number of days for which an abortive medication was consumed throughout the month following the first study visit. The visual analogue scale (VAS) was used to reliably measure the severity of each headache attack. VAS is a Likert-type 10 cm scale, on which 0 (right side of the scale) represents no pain and 10 (left side) shows maximal pain. Mild, moderate, and severe pain indicate a VAS score of 1-3, 4-7, and 8-10, respectively. At the second study visit (30 days after first study visit), headache diaries were retrieved from participants and blood samples were drawn from cases. For the purpose of this study, blood samples were collected from EM cases at least 72 h after their most recent attack to be more representative of the inter-ictal phase of migraine. For CM cases, it was not feasible to collect this blood sample between attacks.

### Blood collection and biochemical tests for assessment of biomarkers

A total volume of 10 mL of peripheral blood (serum) was collected from each participant on abovementioned dates and subsequently divided in 10 microtubes that were stored in -20 °C freezers, and 18 microtubes that were kept in -80 °C freezers. Samples were not stored in freezers for more than 1 year. A part of the blood sample was also isolated as dried blood spots on Whatman^TM^ 903 protein saver cards (Global Life Sciences Solutions USA LLC, Marlborough, MA). The cards were transiently kept at room temperature in the dark for three hours and then stored in a 4 °C fridge. At the time of sampling, participants were asked if they are currently experiencing a headache or not.

All serum samples were sent to the biochemistry laboratory of Sina Hospital, Tehran, Iran. Specimens were analyzed for PGE2 (standard curve range: 2-600 ng/L; sensitivity: 1.28 ng/L), VEGF (standard curve range: 20-6000 ng/L; sensitivity: 10.42 ng/L), NGF (standard curve range: 7-1500 pg/mL; sensitivity: 3.48 pg/mL), and BDNF (standard curve range: 0.05-10 ng/mL; sensitivity: 0.01 ng/mL) using commercial enzyme-linked immunosorbent assay (ELISA) kits from Bioassay Technology Laboratory (Shanghai Korain Biotech Co., Ltd, Shanghai, China) and Crystal day Biotech (Shanghai Crystal day Biotech Co., Ltd., Shanghai, China). Serum levels of these biomarkers were measured as per instructions of the manufacturers of the ELISA kits. Serum samples were tested in batches at different timepoints during the study. All assays were carried out in triplicate. The intra-assay and inter-assay coefficients of variation were <8% and <10%, respectively, which are within generally accepted ranges determined by regulatory bodies [28].

### Sample size and statistical analysis

We did not attempt to calculate the sample size a priori and therefore current study sample size was of convenience. Normality was assessed using the Shapiro-Wilk test. Based on the distribution, continuous data were presented as either mean±standard deviation (SD) or median and interquartile range (IQR). Categorical data were presented as frequency and percentages. For categorical variables, inter-group comparisons (EM vs. CM vs. control) were conducted via Chi-square test. The mean or median values of continuous variables (e.g., serum levels of PGE2, VEGF, NGF, and BDNF) were compared between groups through ANOVA or Kruskal-Wallis test, respectively, and the Dunn-Bonferroni post hoc test was conducted for pairwise comparisons. To adjust for potential confounders, multivariable regression analyses were carried out to model peripheral levels of NGF, BDNF, PGE2, and VEGF as outcome variables. Diagnosis of migraine (EM or CM) or control was the main predictor variable in the model (categorical variable). Age, sex, and BMI were entered as covariates in the model. For EM vs. CM models, headache frequency was dropped from model as it was collinear with the EM/CM diagnosis (variance inflation factor>5). We did not have data on other potential confounders such as comorbidities not designated in the exclusion criteria. Outcome variables were transformed accordingly to minimize non-normal distribution of residuals. Due to persistent heteroscedasticity in linear regression models for all biomarkers, quantile regression was used to model median (quantile=0.5) levels of biomarkers, as opposed to mean levels in linear regression [29, 30]. As a sensitivity analysis, we assessed the effect of prophylactic/abortive drug usage (yes/no to usage of each drug class; categorical variable) if median levels of the biomarker differed significantly in EM vs. CM models adjusted for age, sex, and BMI. Drug classes that had been consumed by at least 10 migraine patients and were significantly associated with levels of biomarkers in bivariable analysis (Mann-Whitney U test) were entered in model(s) for sensitivity analysis. Correlations between serum levels of biomarkers and frequency of headaches was assessed by Pearson or Spearman correlation tests, and correlation coefficients (r and ρ for Pearson and Spearman, respectively) were also calculated. Analyses were all carried out in SPSS 26 (IBM Armonk, NW, US). In all analyses, the level of statistical significance was set at α = 0.05.

## Results

### Baseline characteristics

Eighty-nine subjects (20% males) were enrolled in our study. Thirty-six patients were classified as CM and 23 patients were labeled as EM. Also, 30 healthy controls were evaluated. Table 1 presents the demographics and baseline characteristics of the subjects. Of note, 75% of the migraine patients and 85% of the control subjects were married. Approximately 45% and 55% of the migraineurs and the controls had completed high school, respectively. In addition, about half of the non-headache individuals and 40% of migraine patients had university education (majority not having post-graduate education). Among men, the majority (>80%) were either self-employed or an employee, and most of the women (>75%) were housewives.

**Table 1:** Demographics and baseline characteristics of patients

Variable	Episodic migraine (*n*=23)	Chronic migraine (*n*=36)	Controls (*n*=30)	p-value
Gender *				
- Female	22 (95.65%)	27 (75%)	22 (73.33%)	0.087
- Male	1 (4.34%)	9 (25%)	8 (26.66%)	
Age**	38±9	39±8	41±8	0.509
BMI**	25.24±4.38	26.65±4.37	24.88±3.70	0.178

* Presented as number (%). **Presented as mean±SD

### Headache characteristics

As expected, in CM group, headache was present in 25.74±5.03 days of month while in the EM group, this rate was 8.78±3.26 days (*p*<0.001). The duration of headache during attacks was 19.33±12.30 and 15.48±15.65 h in CM and EM groups, respectively. The VAS scores for severity of headache were also 7.42±2.41 and 7.37±1.82 in CM and EM groups, respectively. No significant difference was observed between the two groups in terms of attack duration and severity (p-values: 0.296 and 0.936, respectively). On the other hand, CM patients were found to consume abortive medications more frequently than EM patients; 14.42±10.25 days/month vs. 6.00±4.17 days/month, respectively (*p*<0.001). Of note, study subjects were consuming abortive (including triptans, ergotamine derivative, and nonsteroidal anti-inflammatory drugs (NSAIDs)) and/or prophylactic medications (including propranolol, tricyclic antidepressants (TCAs), selective serotonin reuptake inhibitors (SSRIs), and serotonin-norepinephrine reuptake inhibitors (SNRIs)) at baseline, and continued to consume these medications after the intervention. Supplementary Table 1 presents a list of types of abortive and prophylactic medications consumed by study patients, and the number and percentage of EM and CM patients taking these medications. A significantly higher percentage of EM patients consumed NSAIDs compared to CM patients (16/23 (69.6%) vs. 14/36 (38.9%); *p*=0.022). Proportions of EM and CM patients consuming other drug classes were not significantly different.

### Comparison of biomarkers between patients with chronic migraine and episodic migraine and controls

**Fig. 1:**
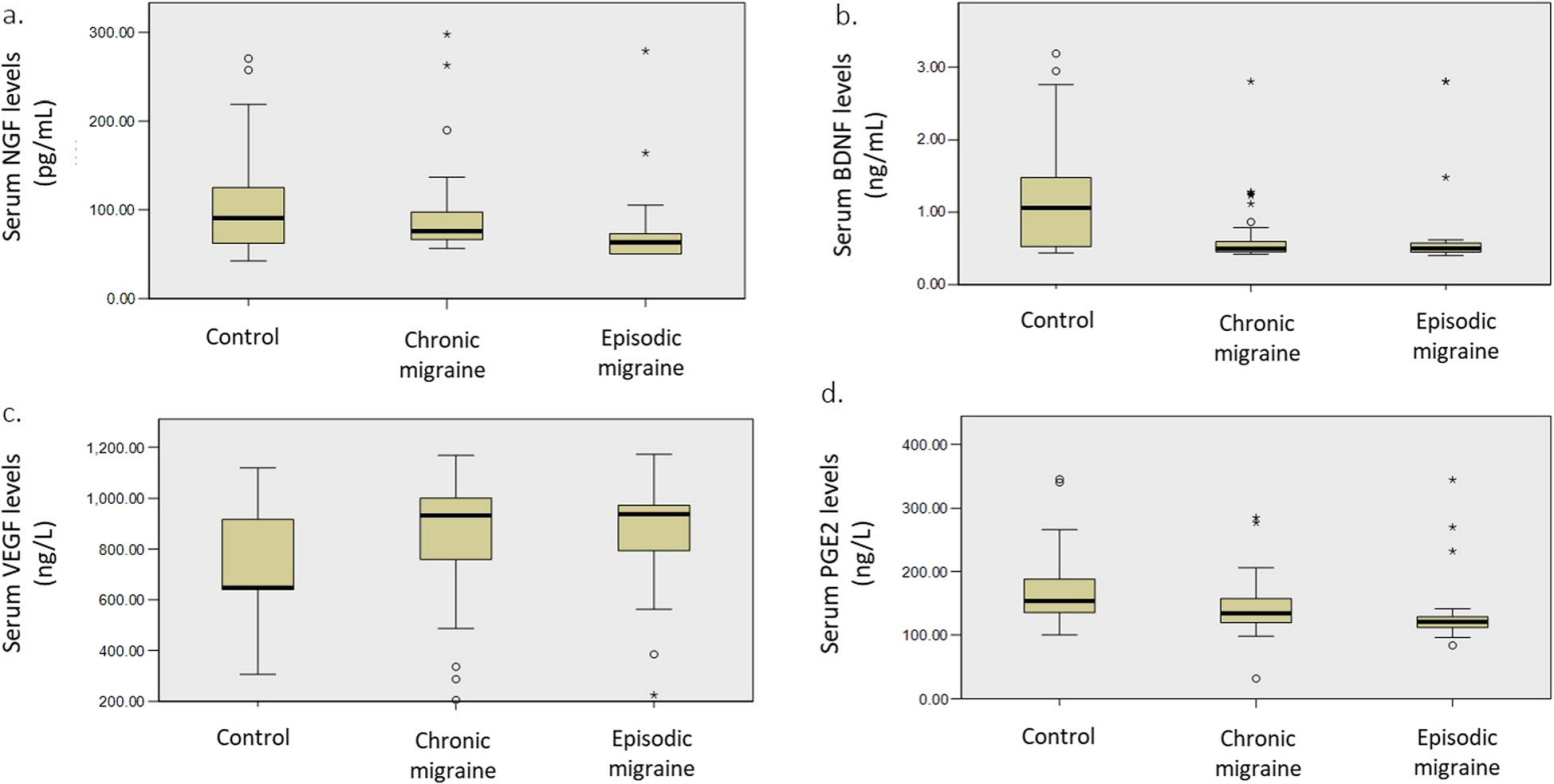
Serum levels of NGF, BDNF, VEGF and PGE2 in EM and CM patients, and controls. **A** NGF (**B**) BDNF (**C**) VEGF (**D**) PGE2; NGF: nerve growth factor; BDNF: brain-derived neurotrophic factor (BDNF); VEGF: vascular endothelial growth factor; PGE2: prostaglandin E2; EM: episodic migraine; CM: chronic migraine

Distribution of measured serum levels of NGF, BDNF, VEGF and PGE2 in EM, CM, and control subjects are visualized in Fig. 1(A-D). Comparisons of the aforementioned biomarkers between the three groups of study participants are presented in Table 2. NGF values were significantly lower among EM patients as opposed to CM patients and controls (63.11±24.56 vs. 75.88±31.89 and 90.58±70.25, respectively). Despite NGF levels being lower in CM patients than in controls, the difference was not statistically significant. BDNF levels were significantly lower in CM and EM patients compared to controls (CM vs. control: 0.49±0.14 vs. 1.05±0.96; EM vs. control: 0.49±0.13 vs. 1.05±0.96) but almost identical between CM and EM patients. PGE2 levels were lower in EM and CM patients as opposed to controls (120.71±19.47 and 134.16±38.57 for EM and CM, respectively, and 153.50±55.69 for control group). However, the difference was only statistically significant for the EM vs. control comparison. In addition, although levels of PGE2 were higher in CM patients compared to EM subjects (134.16±38.57 vs. 120.71±19.47), the difference failed to reach statistical significance. Conversely, VEGF levels corresponding to CM and EM patients were revealed to be higher than that of controls; 932.56±301.35 and 937.41±230.23, respectively, vs. 646.50±292.50. Despite the significant difference among groups in this regard, adjusted pairwise comparisons failed to confirm the significant difference between EM, CM and control-specific serum VEGF levels.

**Table 2:** Serum levels of NGF, BDNF, VEGF and PGE2 biomarkers in study groups

Biomarker	Episodic migraine (*n*=23)	Chronic migraine (*n*=36)	Controls (*n*=30)	*P-*value^¶^
	Median (IQR)	Median (IQR)	Median (IQR)	
NGF	63.11 (24.56) ^*, #^	75.88 (31.89) ^*^	90.58 (70.25) ^#^	0.009
BDNF	0.49 (0.13) ^*^	0.49 (0.14) ^#^	1.05 (0.96) ^*, #^	<0.001
VEGF	937.41 (230.23)	932.56 (301.35)	646.50 (292.50)	0.046
PGE2	120.71 (19.47)^*^	134.16 (38.57)	153.50 (55.69)^*^	0.001

¶: Across group comparison (Kruskal-Wallis); *, #: significant adjusted *P-*value for pairwise-comparison (Dunn-Bonferroni); NGF: nerve growth factor; BDNF: brain-derived neurotrophic factor; VEGF: vascular endothelial growth factor; PGE2: prostaglandin E2

In multivariable regression analysis (quantile regression; Table 3), an EM diagnosis independently predicted significantly lower median levels of NGF compared to being in the control group (P-value= 0.003) or having a CM diagnosis (P-value= 0.042). NGF levels were not significantly different in CM vs. control regression analysis (P-value= 0.156). Moreover, an EM or CM diagnosis (versus control) independently predicted significantly lower median levels of BDNF compared to being a control (P-value= <0.001). However, BDNF levels were not different between EM and CM patients when adjusted for age, sex, and BMI (P-value=0.715). In addition, an EM diagnosis (versus control) significantly predicted median levels of PGE2 (P-value= 0.011). Although PGE2 levels were lower in EM patients compared to CM patients, and lower in CM patients compared to controls, the differences failed to reach statistical significance in adjusted regression models (P-value= 0.086 and 0.163, respectively). Also, an EM or CM diagnosis (versus control) independently predicted significantly higher median levels of VEGF (*P*<0.001 for both comparisons). Median levels of VEGF were not different between EM and CM patients in adjusted regression models (P-value= 0.859). In sensitivity analysis, having an CM diagnosis (as opposed to EM) independently predicted significantly higher median levels of NGF, when adjusted for age, sex, BMI, NSAID usage, and beta blocker usage (P-value= 0.017).

**Table 3:** Parameter estimates in multivariable analysis of factors associated with peripheral levels of NGF, BDNF, PGE2, and VEGF

Characteristic	Coefficient	P-value	95% CI for coefficient	
			Lower bound	Upper Bound
NGF model: EM diagnosis (vs. control)	-31.360	0.003	-51.683	-11.038
NGF model: EM diagnosis (vs. CM)	-17.148	0.042	-33.618	-0.678
NGF model: CM diagnosis (vs. control)	-12.994	0.156	-31.052	5.064
BDNF model: EM diagnosis (vs. control)	-0.583	<0.001	-0.715	-0.450
BDNF model: CM diagnosis (vs. control)	-0.572	<0.001	-0.690	-0.454
BDNF model: CM diagnosis (vs. EM)	0.017	0.715	-0.077	0.111
PGE2 model: EM diagnosis (vs. control)	-29.284	0.011	-51.781	-6.788
PGE2 model: CM diagnosis (vs. EM)	15.324	0.086	-2.251	32.899
PGE2 model: CM diagnosis (vs. control)	-14.163	0.163	-34.152	5.827
VEGF model: EM diagnosis (vs. control)	258.626	<0.001	168.119	349.132
VEGF model: CM diagnosis (vs. control)	261.613	<0.001	181.191	342.035
VEGF model: CM diagnosis (vs. EM)	13.837	0.859	-141.643	169.318
Sensitivity NGF model: CM diagnosis (vs. EM)	18.855	0.017	3.455	34.256

*Quantile regression (quantile= 0.5 [median]). All models adjusted for age (continuous), sex (binary), and BMI (continuous). EM/CM/control diagnosis as categorical variable

**NGF: nerve growth factor; BDNF: brain-derived neurotrophic factor; PGE2: prostaglandin E2; VEGF: vascular endothelial growth factor; CI: confidence interval; EM: episodic migraine; CM: chronic migraine

*** Sensitivity model also adjusted for non-steroidal anti-inflammatory drug usage (binary) and beta blocker drug usage (binary)

**Fig. 2:**
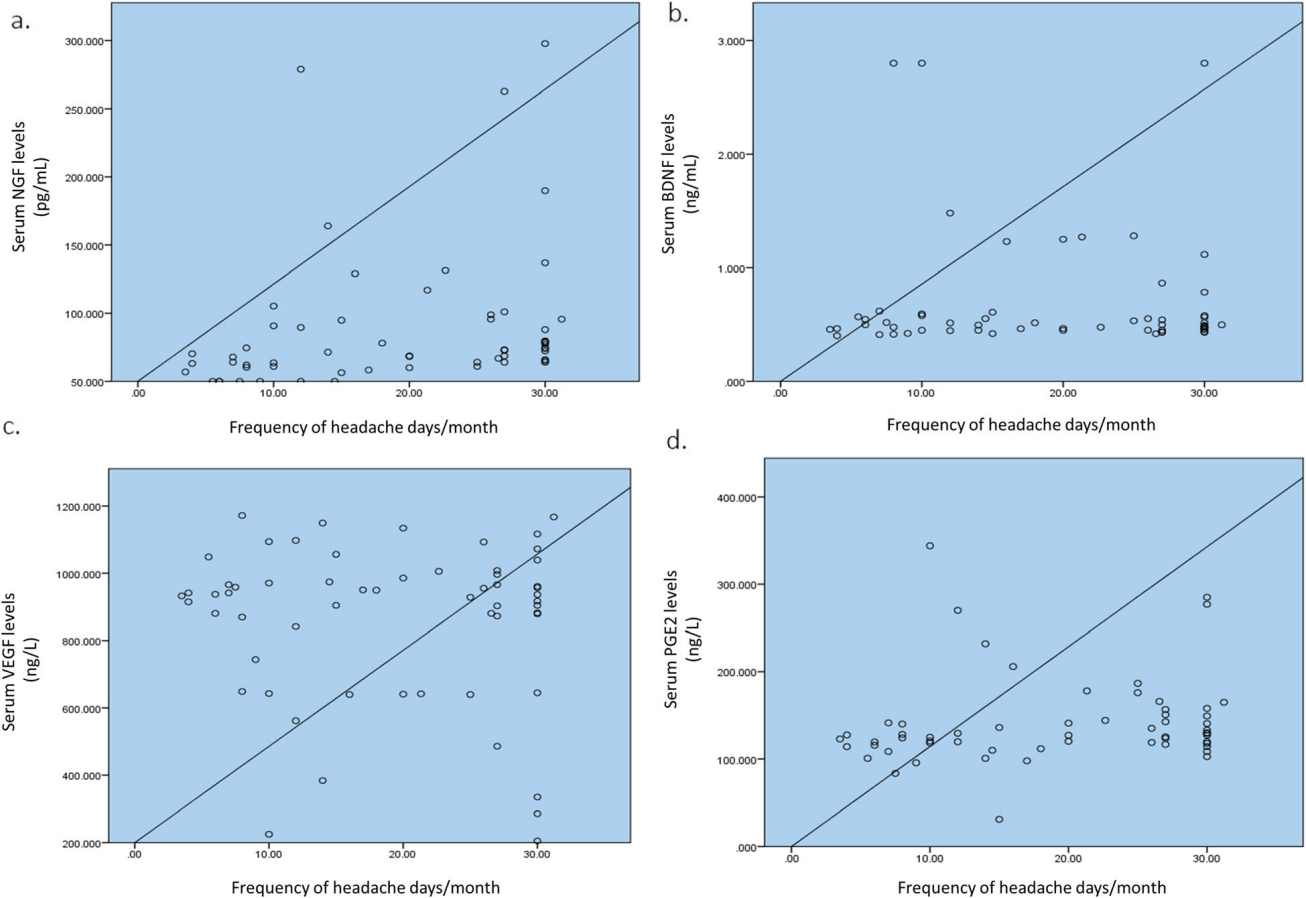
Correlation between headache frequency and Serum levels of NGF, BDNF, VEGF and PGE2. **A** NGF (**B**) BDNF (**C**) VEGF (**D**) PGE2; NGF: nerve growth factor; BDNF: brain-derived neurotrophic factor (BDNF); VEGF: vascular endothelial growth factor; PGE2: prostaglandin E2

Correlations between serum levels of NGF, BDNF, VEGF, and PGE2 in persons with migraine disease (combined EM and CM patients) and frequency of their headaches (i.e., number of headache days in each month) are illustrated in Fig. 2(A-D). A higher frequency of headache days correlated positively with higher levels of serum NGF (ρ = 0.476; p-value<0.001) and PGE2 (ρ = 0.286; p-value=0.028), while serum VEGF and BDNF levels did not correlate significantly with number of headache days/month in migraine patients (BDNF: ρ = 0.037 and p-value=0.778; VEGF: ρ= -0.025 and p-value=0.850).

## Discussion

With the aim to improve our understanding of the underlying mechanisms of migraine as a highly prevalent condition around the world, we sought to identify potential biomarkers involved in the pathogenesis of migraine. In line with this aim, we focused on the serum levels of PGE2, VEGF, NGF and BDNF in patients suffering from CM or EM. In addition to the detected lower levels of NGF, BDNF and PGE2 among EM patients compared to controls, NGF was the only biomarker in our study that showed significantly different (i.e., lower) serum levels in EM patients than that of CM patients, while none of the remaining three biomarkers investigated in our case control study were able to discern between CM and EM (although difference in PGE2 levels between EM and CM patients trended towards significance). Moreover, our results indicated that higher NGF and PGE2 serum levels have a moderate and weak positive correlation with headache frequency in migraine patients, respectively, while no significant correlation was replicated for BDNF and VEGF.

NGF is a neurotrophin mainly found in the limbic system [31]. It is recognized to be involved in cognition, mood, protection of neurons, neuroplasticity and response to stress mechanisms [32, 33]. Peripheral expression of NGF has also been associated with nociceptive sensitization; pain is conveyed from trigeminal ganglion to trigeminal nucleus caudalis through TRPV1 [34, 35]. Our study demonstrated that peripheral blood NGF levels are significantly lower in EM patients compared to healthy controls and CM subjects. A study by Blandini et al. evaluating migraine patients, cluster headache patients, and controls corroborated our findings, as they showed a reduction in plasma and platelet levels of NGF in migraine patients in comparison to controls [36]. In contrast, a study by Jang et al. on patients with CM and controls revealed that levels of NGF and other neuropeptides such as CGRP and substance P showed elevations in persons with migraine disease, a positive correlation was noted between NGF and these other neuropeptides, and levels of NGF and the aforementioned neuropeptides significantly correlated with intensity of pain [37]. In a study conducted by Sarchielli et al. on patients with chronic daily headache with history of migraine and controls, elevated cerebrospinal fluid (CSF) levels of NGF were observed in patients with migraine [38]. The same research group also demonstrated that CSF levels of NGF positively correlate with headache frequency, in line with our current study findings, but do not correlate with VAS scores representative of headache severity [38, 39]. Another study by Sarchielli et al. also showed that CSF levels of NGF and glutamate are significantly higher in migraine patients compared to controls [35]. Of note, a study by Martins et al. also showed no difference between migraine patients and controls in terms of plasma levels of NGF [18]. Nevertheless, the inconsistencies in the literature regarding the levels of NGF in patients with migraine warrants the need for further investigations to reach a more definite conclusion.

BDNF is the most abundant neurotrophin found in different compartments of the nervous system [40]. BDNF is involved in pain signaling and regulation, in addition to the roles in neuronal development and differentiation [36, 41, 42]. With regards to pain, BDNF exerts a paradoxical, dose-dependent effect where low doses lead to hyperalgesia, while high doses result in analgesia [43]. Its expression is associated with CGRP in trigeminal ganglion neurons. Researchers have suggested that these associations of BDNF may play a significant role in modulating susceptibility of patients to migraine [44]. Our study showed that EM and CM patients have significantly lower levels of BDNF compared to healthy controls. We postulate that this finding might be explained by the hyperalgesic effect of BDNF in lower doses. Martins et al. and Blandini et al. have confirmed our BDNF findings in CM patients (compared to controls) [18, 36]. However, most other studies in the literature have indicated the opposite. For example, Sarchielli et al. have indicated that CSF levels of BDNF are significantly higher in persons with migraine disease, CSF levels of NGF and BDNF positively correlate with each other, and BDNF levels in the CSF correlate with number of headache days in each month (although correlation was weaker compared to NGF and headache frequency) but not with headache severity [35, 39]. Regarding their latter finding, our current study did not find a significant correlation between headache frequency and serum BDNF levels. In 2010, Tanure and colleagues demonstrated that BDNF serum levels are significantly elevated during migraine attacks [42]. Another study also revealed higher levels of BDNF in migraine attacks compared to headache-free period and tension-type headaches [45]. Regarding our finding of similar BDNF levels between CM and EM patients, we were not able to identify studies that have reached a similar conclusion as no study to our knowledge has reported on findings regarding direct comparison of BDNF levels between EM and CM patients. Regardless of the inverse or direct relationship between BDNF levels and propensity for migraine, these various levels of evidence emphasize the role of BDNF in nociceptive pathways. One explanation for the reduced levels of both neurotrophins (NGF and BDNF) in our study could be that a subgroup of the EM and CM patients had concomitant undiagnosed depression and therefore were not receiving antidepressants. It has been previously propounded that patients suffering from depression may have decreased levels of NGF and BDNF, and fluoxetine might increase those levels [46].

In pathophysiology of migraine, release of vasoactive substances by meningeal and brain mast cells may possibly play a pivotal role because this can trigger trigeminovascular mechanisms leading to development of pain [47]. VEGF is one of these substances which comprises an array of glycoproteins that contribute significantly towards cellular protection and angiogenesis. Additionally, these glycoproteins are regarded as potential proinflammatory cytokines [48, 49]. Our study revealed higher peripheral levels of VEGF in both EM and CM patients compared to healthy controls. Rodriguez-Osorio X et al. [50] have also reported that VEGF levels are significantly higher in patients with episodic migraines compared to controls. One proposed rationale for the increased VEGF levels in persons with migraine disease could be that this response is of a compensatory manner.[50] Moreover, it could herald the onset of chronic endothelial dysfunction in apparently healthy individuals. In addition, SSRIs have been suggested to increase levels of VEGF both centrally (i.e., in the hippocampus and dentate gyrus) and peripherally in the blood, [51, 52] and this could have been the case for our participants who were taking SSRIs or even SNRIs and TCAs. On the other hand, significant reductions in VEGF levels during the interictal period have been reported by Michalak et al. [17] It is important to further investigate the association between VEGF and migraine in future studies. The importance of this association could be due to higher rates of cardiovascular accidents in patients suffering from migraine; increased risk of ischemic stroke in women and increased risk of myocardial infarction in men with migraine have been reported in the literature [53, 54]. Some authors have speculated that this relationship is due to endothelial changes reflected in alterations of factors such as VEGF.

PGE2, as a member of the prostaglandin family, has been reported to be involved in underlying mechanisms of pain in migraine [13]. The pain induced by PGE2 might possibly stem from the activation of TRPV1 receptors [55]. Some studies have shown that infusion of PGE2 leads to headache in 83-100% of patients while this rate after prostaglandin F2-alpha infusion has been only 17% [56–58]. However, our study showed that serum PGE2 levels are significantly lower in EM patients compared to controls, and while they are also lower in CM patients in contrast to controls, the difference is not statistically significant. Sarchielli et al. showed that internal jugular venous blood levels of PGE2 maximize within two hours of headache onset, plateau until hours 4-6 post-headache onset, and then decline [59]. Mohammadian and colleagues showed that PGE2 levels in saliva and nasal lavage samples of migraine patients during the inter-ictal phase are comparable to that of controls [60] Tuca et al. showed when saliva is collected during a headache attack from migraine patients, PGE2 levels are significantly higher than that of samples corresponding to the period between attacks [61]. They also showed that PGE2 saliva levels declined in persons with migraine disease who consumed calcium channel blockers (CCBs) for two months, as opposed to those migraine patients who were given placebo, and this could explain the lower levels of PGE2 in our migraine study subjects who were taking CCBs at baseline. Similarly, Li et al. demonstrated that in migraine patients, cyclooxygenase-2 (COX-2; enzyme that gives rise to PGE2) levels are significantly higher in the attack period as opposed to the headache-free period, and comparable between controls and migraine patients during the inter-ictal phase [62]. As close to 40% and 70% of our study participants in the CM and EM groups were taking NSAIDS, this could have affected PGE2 levels, especially if they consumed a selective COX-2 inhibitor in close proximity to the blood draw.

In our study, NGF and PGE2 serum levels positively correlated with headache frequency in migraine patients, while serum levels for BDNF and VEGF did not show such significant correlations. We were not able to find any study in the literature assessing the correlation between headache frequency and levels of *in vivo* PGE2 and VEGF, and we found only two studies that evaluated the correlation between CSF levels (not serum) of NGF and BDNF and number of headache days [38, 39]. Therefore, it is difficult to draw any meaningful conclusion. Nevertheless, based on our current findings and Sarchielli et al.’s studies, [38, 39] there might be a possibility that higher levels of NGF and PGE2 could herald the “transformation” of EM to CM. If this is true, one could possibly consider a more prominent role of inflammation in migraine evolution/”chronification”. In 2018, we showed that serum levels of CRP and tumor necrosis factor alpha (TNF-α) did not correlate with frequency of migraine attacks [63]. Most recently, however, we showed that serum levels of proinflammatory biomarkers including CRP, TNF-α, interleukin-6, NO, and malondialdehyde positively correlated with number of migraine headache days per month [11, 12]. These recent findings of our research group and the postulated role for inflammation in CM support the role of inflammation in migraine evolution [64].

For biomarkers in our study with significantly lower levels in EM and/or CM patients compared to controls (NGF, BDNF, and PGE2), one common possible explanation, at least for EM patients perhaps, could be that the minimum 72-hour lag between headache onset and blood collection may have been too long, and resulted in sizeable drops from peak levels during attacks. This may have also explained the comparability of serum levels of BDNF, VEGF, and PGE2 between EM and CM patients. One other explanation could be that although migraine patients and controls were age and sex-matched, they may not have been matched for silent proinflammatory states (for example, conditions with heightened levels of C-reactive protein (CRP) and other proinflammatory cytokines) that might influence NGF, BDNF, VEGF, and PGE2 levels. A combination of timing of blood draw in reference to most recent headache attack and confounding non-diagnosed proinflammatory comorbidities might possibly explain the significant difference of serum levels of NGF between EM and CM patients in our study. Finally, difference in study populations and laboratory assays used for measuring the biomarkers could have biased our results towards the opposite direction of some of the previously mentioned findings in the literature.

We believe that our study has several strengths; first, classification errors for EM and CM were at the minimum as diagnosis of eligible participants was confirmed by a neurologist specialized in headaches. Second, patients previously diagnosed with MOH were not included, hence decreasing confounding bias of medication effects on biomarker levels. Third, the one-month length of follow up for documenting headache characteristics of migraine patients was relatively robust. We also acknowledge that our study faced several limitations; the case-control nature of the study prohibited us from drawing conclusions about causality and directionality between migraine and NGF, BDNF, VEGF, and PGE2 peripheral blood levels. Our study was not powered to detect pre-defined differences between levels of biomarkers in EM and CM patients, and healthy controls as no formal sample size calculation was conducted. As mentioned above, we were not able to control for multiple confounding factors that might have influenced levels of biomarkers. It was not feasible for us to measure the concurrent CSF levels of our investigated biomarkers, so it should be clarified that peripheral levels of biomarkers in our study participants might not reflect changes in their central nervous systems. Distribution of females was different between EM and CM patients, although the difference was not statistically significant, and this may have masked any possible gender effect modification on serum biomarker levels.

## Conclusions

Our study showed that peripheral blood levels of NGF, BDNF, VEGF, and PGE2 are significantly different in EM and/or CM patients compared to healthy controls, and peripheral NGF levels, and to a lesser degree serum PGE2 levels, may be able to differentiate between EM and CM patients. Further prospective cohort studies with larger sample sizes can advance our knowledge of the role of these biomarkers and other candidates in migraine pathophysiology, and possibly facilitate the use of these biomarkers for modification of current diagnostic, prognostic, and therapeutic approaches towards migraine headaches. Specifically, more work is needed in the area of differentiating EM and CM patients via candidate biomarkers to identify individuals who could possibly benefit from earlier therapeutic interventions.

## Supplementary information

**Additional file 1.**

## Data Availability

The datasets used and/or analyzed during the current study are available from the corresponding author on reasonable request.

## Abbreviations

ANOVA: Analysis of variance
BMI: Body mass index
BDNF: Brain-derived neurotrophic factor
CGRP: Calcitonin gene-related peptide
CCB: Calcium channel blocker
CSF: Cerebrospinal fluid
CM: Chronic migraine
CSD: Cortical spreading depression
CRP: C-reactive protein
COX-2: Cyclooxygenase-2
ELISA: Enzyme-linked immunosorbent assay
EM: Episodic migraine
GBD: Global burden disease
IQR: Interquartile range
MOH: Medication overuse headache
NIMAD: National Institute for Medical Research Development
NGF: Nerve growth factor
NO: Nitric oxide
NSAID: Nonsteroidal anti-inflammatory drug
PACAP: Pituitary adenylate cyclase-activating peptide
PGE2: Prostaglandin E2
SSRI: Selective serotonin reuptake inhibitor
SNRI: Serotonin-norepinephrine reuptake inhibitor
SD: Standard deviation
TXA2: Thromboxane A2
TRPV1: Transient receptor potential vanilloid receptor 1
TCA: Tricyclic antidepressants
TNF-α: Tumor necrosis factor alpha
VEGF: Vascular endothelial growth factor
VAS: Visual analogue scale

## References

[1] Agosti R (2018). Migraine Burden of Disease: From the Patient’s Experience to a Socio-Economic View. Headache: The Journal of Head and Face Pain.

[2] Leonardi M, Raggi A (2019). A narrative review on the burden of migraine: when the burden is the impact on people’s life. J Headache Pain.

[3] Steiner TJ, Stovner LJ, Vos T (2018). Migraine is first cause of disability in under 50s: will health politicians now take notice?. J Headache Pain.

[4] Global, regional, and national burden of migraine and tension-type headache, 1990-2016: a systematic analysis for the Global Burden of Disease Study 2016.Lancet Neurol17:954–976. 10.1016/s1474-4422(18)30322-3 10.1016/S1474-4422(18)30322-3 PMC6191530 30353868

[5] Steiner TJ, Stovner LJ, Jensen R (2020). Migraine remains second among the world’s causes of disability, and first among young women: findings from GBD2019. J Headache Pain.

[6] Ghorbani Z, Rafiee P, Haghighi S (2021). The effects of vitamin D3 supplementation on TGF-β and IL-17 serum levels in migraineurs: post hoc analysis of a randomized clinical trial. Journal of Pharmaceutical Health Care and Sciences.

[7] Ashina M, Terwindt GM, Al-Karagholi MA-M (2021). Migraine: disease characterisation, biomarkers, and precision medicine. The Lancet.

[8] Headache Classification Committee of the International Headache Society (IHS) The International Classification of Headache Disorders, 3rd edition. Cephalalgia 38:1–211. 10.1177/0333102417738202 10.1177/0333102417738202 29368949

[9] Ashina M (2020). Migraine. N Engl J Med.

[10] Dodick DW (2018). A Phase-by-Phase Review of Migraine Pathophysiology. Headache: The Journal of Head and Face Pain.

[11] Togha M, Razeghi Jahromi S, Ghorbani Z (2019). An investigation of oxidant/antioxidant balance in patients with migraine: a case-control study. BMC Neurol.

[12] Togha M, Jahromi SR, Ghorbani Z et al (2020) Evaluation of Inflammatory State in Migraineurs: A Case-control Study. Iranian Journal of Allergy, Asthma and Immunology 19. 10.18502/ijaai.v19i(s1.r1).2864 10.18502/ijaai.v19i(s1.r1).2864 32534515

[13] Antonova M, Wienecke T, Olesen J, Ashina M (2013). Prostaglandins in migraine: update. Curr Opin Neurol.

[14] Ghorbani Z, Togha M, Rafiee P (2019). Vitamin D in migraine headache: a comprehensive review on literature. Neurol Sci.

[15] Zhang H, Zhang X, Zong D (2021). miR-34a-5p up-regulates the IL-1β/COX2/PGE2 inflammation pathway and induces the release of CGRP via inhibition of SIRT1 in rat trigeminal ganglion neurons. FEBS Open Bio.

[16] Jacobs B, Dussor G (2016). Neurovascular contributions to migraine: Moving beyond vasodilation. Neuroscience.

[17] Michalak S, Kalinowska-Lyszczarz A, Wegrzyn D (2017). The Levels of Circulating Proangiogenic Factors in Migraineurs. Neuromol Med.

[18] Martins LB, Duarte H, Ferreira AVM (2015). Migraine is associated with altered levels of neurotrophins. Neurosci Lett.

[19] Oterino A, Toriello M, Valle N (2010). The Relationship Between Homocysteine and Genes of Folate-Related Enzymes in Migraine Patients. Headache: The Journal of Head and Face Pain.

[20] Mottaghi T, Khorvash F, Askari G (2013). The relationship between serum levels of vitamin D and migraine. Journal of research in medical sciences: the official journal of Isfahan University of Medical Sciences.

[21] Ghorbani Z, Togha M, Rafiee P (2020). Vitamin D3 might improve headache characteristics and protect against inflammation in migraine: a randomized clinical trial. Neurol Sci.

[22] Ghorbani Z, Rafiee P, Fotouhi A (2020). The effects of vitamin D supplementation on interictal serum levels of calcitonin gene-related peptide (CGRP) in episodic migraine patients: post hoc analysis of a randomized double-blind placebo-controlled trial. J Headache Pain.

[23] Burstein R, Noseda R, Borsook D (2015). Migraine: Multiple Processes, Complex Pathophysiology. The Journal of Neuroscience.

[24] Silberstein SD, Holland S, Freitag F (2012). Evidence-based guideline update: Pharmacologic treatment for episodic migraine prevention in adults. Neurology.

[25] Hepp Z, Bloudek LM, Varon SF (2014). Systematic Review of Migraine Prophylaxis Adherence and Persistence. Journal of Managed Care Pharmacy.

[26] Blumenfeld AM, Bloudek LM, Becker WJ (2013). Patterns of Use and Reasons for Discontinuation of Prophylactic Medications for Episodic Migraine and Chronic Migraine: Results From the Second International Burden of Migraine Study (IBMS-II). Headache: The Journal of Head and Face Pain.

[27] Razeghi Jahromi S, Abolhasani M, Ghorbani Z (2018). Bariatric Surgery Promising in Migraine Control: a Controlled Trial on Weight Loss and Its Effect on Migraine Headache. Obes Surg.

[28] Guidance Document (2021) : Bioanalytical Method Validation Guidance for Industry. FDA. MAY 2018. Accessed October 7,

[29] Stuginski-Barbosa J, Dach F, Bigal M, Speciali JG (2012). Chronic Pain and Depression in the Quality of Life of Women With Migraine – A Controlled Study. Headache: The Journal of Head and Face Pain.

[30] Bottai M, Cai B, McKeown RE (2010). Logistic quantile regression for bounded outcomes. Stat Med.

[31] Cirulli F, Alleva E (2009). The NGF saga: From animal models of psychosocial stress to stress-related psychopathology. Front Neuroendocr.

[32] Shu X-Q, Mendell LM (1999) Neurotrophins and hyperalgesia. Proceedings of the National Academy of Sciences 96:7693. 10.1073/pnas.96.14.7693 10.1073/pnas.96.14.7693 PMC33603 10393882

[33] Yeh Y-W, Kuo S-C, Chen C-Y (2015). Harm avoidance involved in mediating the association between nerve growth factor (NGF) gene polymorphisms and antidepressant efficacy in patients with major depressive disorder. J Affect Disord.

[34] Shimizu T, Shibata M, Suzuki N (2011). Migraine: Advances in the pathophysiology and treatment. Rinsho Shinkeigaku.

[35] Sarchielli P, Mancini ML, Floridi A (2007). Increased Levels of Neurotrophins Are Not Specific for Chronic Migraine: Evidence From Primary Fibromyalgia Syndrome. The Journal of Pain.

[36] Blandini F, Rinaldi L, Tassorelli C (2006). Peripheral Levels of BDNF and NGF in Primary Headaches. Cephalalgia.

[37] Jang M-U, Park J-W, Kho H-S (2011). Plasma and saliva levels of nerve growth factor and neuropeptides in chronic migraine patients. Oral Dis.

[38] Sarchielli P, Alberti A, Floridi A, Gallai V (2001). Levels of nerve growth factor in cerebrospinal fluid of chronic daily headache patients. Neurology.

[39] Sarchielli P, Alberti A, Gallai B (2002). Brain-derived neurotrophic factor in cerebrospinal fluid of patients with chronic daily headache: relationship with nerve growth factor and glutamate levels. J Headache Pain.

[40] LIPSKY RH, MARINI ANNM (2007). Brain-Derived Neurotrophic Factor in Neuronal Survival and Behavior-Related Plasticity. Ann N Y Acad Sci.

[41] Thompson SWN, Bennett DLH, Kerr BJ et al (1999) Brain-derived neurotrophic factor is an endogenous modulator of nociceptive responses in the spinal cord. Proceedings of the National Academy of Sciences 96:7714. 10.1073/pnas.96.14.7714 10.1073/pnas.96.14.7714 PMC33607 10393886

[42] Tanure MTA, Gomez RS, Hurtado RCL (2010). Increased serum levels of brain-derived neurotropic factor during migraine attacks: a pilot study. J Headache Pain.

[43] Sordyl J, Małecka-Tendera E, Sarecka-Hujar B, Kopyta I (2020). Headache in Children: Selected Factors of Vascular Changes Involved in Underlying Processes of Idiopathic Headaches. Children (Basel, Switzerland).

[44] Lemos C, Mendonça D, Pereira-Monteiro J (2010). BDNF and CGRP interaction: Implications in migraine susceptibility. Cephalalgia.

[45] Fischer M, Wille G, Klien S (2012). Brain-derived neurotrophic factor in primary headaches. J Headache Pain.

[46] Mondal AC, Fatima M (2019). Direct and indirect evidences of BDNF and NGF as key modulators in depression: role of antidepressants treatment. Int J Neurosci.

[47] Llorián-Salvador M, González-Rodríguez S (2018). Painful Understanding of VEGF. Front Pharmacol.

[48] Ferrara N, Gerber H-P, LeCouter J (2003). The biology of VEGF and its receptors. Nat Med.

[49] Reinders MEJ, Sho M, Izawa A (2003). Proinflammatory functions of vascular endothelial growth factor in alloimmunity. J Clin Investig.

[50] Rodríguez-Osorio X, Sobrino T, Brea D (2012). Endothelial progenitor cells. Neurology.

[51] Takebayashi M, Hashimoto R, Hisaoka K (2010). Plasma levels of vascular endothelial growth factor and fibroblast growth factor 2 in patients with major depressive disorders. J Neural Transm.

[52] Loulergue P, Mir O, Rocheteau P (2016). Sertraline-induced increase in VEGF brain levels and its activity in cryptococcal meningitis. Lancet Infect Dis.

[53] MacClellan LR, Giles W, Cole J (2007). Probable Migraine With Visual Aura and Risk of Ischemic Stroke. Stroke.

[54] Kurth T, Gaziano JM, Cook NR (2007). Migraine and Risk of Cardiovascular Disease in Men. Arch Intern Med.

[55] Moriyama T, Higashi T, Togashi K (2005). Sensitization of TRPV1 by EP1 and IP reveals peripheral nociceptive mechanism of prostaglandins. Mol Pain.

[56] Wienecke T, Olesen J, Oturai PS, Ashina M (2009). Prostaglandin E2 (PGE2) Induces Headache in Healthy Subjects. Cephalalgia.

[57] Wienecke T, Olesen J, Oturai PS, Ashina M (2008) Prostacyclin (epoprostenol) induces headache in healthy subjects.PAIN139 10.1016/j.pain.2008.03.018 18450380

[58] Antonova M, Wienecke T, Olesen J, Ashina M (2011). Pro-inflammatory and vasoconstricting prostanoid PGF2α causes no headache in man. Cephalalgia.

[59] Sarchielli P, Alberti A, Codini M (2000). Nitric Oxide Metabolites, Prostaglandins and Trigeminal Vasoactive Peptides in Internal Jugular Vein Blood During Spontaneous Migraine Attacks. Cephalalgia.

[60] Mohammadian P, Hummel T, Arora C, Carpenter T (2001). Peripheral Levels of Inflammatory Mediators in Migraineurs During Headache-free Periods. Headache: The Journal of Head and Face Pain.

[61] Tuca JO, Planas JM, Parellada RP (1989) Increase in PGE2 and TXA2 in the Saliva of Common Migraine Patients. Action of Calcium Channel Blockers. Headache: The Journal of Head and Face Pain 29:498–501. 10.1111/j.1526-4610.1989.hed2908498.x 10.1111/j.1526-4610.1989.hed2908498.x 2793453

[62] Li C, Zhu Q, He Q (2017). Plasma Levels of Cyclooxygenase-2 (COX-2) and Visfatin During Different Stages and Different Subtypes of Migraine Headaches. Medical science monitor: international medical journal of experimental and clinical research.

[63] Martami F, Razeghi Jahromi S, Togha M (2018). The serum level of inflammatory markers in chronic and episodic migraine: a case-control study. Neurol Sci.

[64] Torres-Ferrús M, Ursitti F, Alpuente A (2020). From transformation to chronification of migraine: pathophysiological and clinical aspects. J Headache Pain.

