# Supplementary material for: Assessment of peripheral biomarkers potentially involved in episodic and chronic migraine: a case-control study with a focus on NGF, BDNF, VEGF, and PGE2
Source: J Headache Pain. 2022 Jan 6;23(1):3. doi: 10.1186/s10194-021-01377-6 (PMC8903594; doi:10.1186/s10194-021-01377-6)
Supplement: Supplementary file 1 — Additional file 1. [file 10194_2021_1377_MOESM1_ESM.docx]

**Supplementary Table 1. Consumption of medications in episodic and chronic migraine groups**

| Medication | Episodic migraine | Chronic migraine | *P-*value |
| --- | --- | --- | --- |
| Beta blocker | 10 (43.5%) | 8 (22.2%) | 0.084 |
| Sodium Valproate | 7 (30.4%) | 7 (19.4%) | 0.333 |
| CCB | 0 (0%) | 2 (5.6%) | 0.250 |
| TCA | 3 (13%) | 11 (30.6%) | 0.123 |
| SSRI | 2 (8.7%) | 8 (22.2%) | 0.177 |
| SNRI | 0 (0%) | 4 (11.1%) | 0.098 |
| Bupropion | 0 (0%) | 2 (5.6%) | 0.250 |
| Benzodiazepine | 0 (0%) | 1 (2.8%) | 0.420 |
| Coenzyme Q10 | 7 (30.4%) | 6 (16.7%) | 0.219 |
| Magnesium | 6 (26.1%) | 6 (16.7%) | 0.381 |
| Sumatriptan/Rizatriptan | 9 (39.1%) | 11 (30.6%) | 0.497 |
| NSAID | 16 (69.6%) | 14 (38.9%) | 0.022 |
| Non-NSAID analgesics | 9 (39.1%) | 7 (19.4%) | 0.097 |
| Miscellaneous analgesics | 0 (0%) | 2 (5.6%) | 0.250 |
| Opioid | 0 (0%) | 3 (8.3%) | 0.155 |
| Oral corticosteroids | 0 (0%) | 1 (2.8%) | 0.420 |
| ASA | 0 (0%) | 1 (2.8%) | 0.420 |

All values in table cells presented as number (%). TCA: tricyclic anti-depressant; SSRI: selective serotonin reuptake inhibitor; SNRI: serotonin norepinephrine reuptake inhibitor; NSAID: non-steroidal anti-inflammatory drug; ASA: aspirin; CCB: calcium channel blocker
